# Assessment of violence risk in 440 psychiatric patients in China: examining the feasibility and acceptability of a novel and scalable approach (FoVOx)

**DOI:** 10.1186/s12888-021-03115-3

**Published:** 2021-03-02

**Authors:** Shaoling Zhong, Rongqin Yu, Cornish Robert, Xiaoping Wang, Seena Fazel, FoVOx group, Chen Chen, Chun Li, Dengke Zhang, Jun Wang, Shenci Li, Simei Zhang, Xiaomin Zhu, Ying Lv, Zhimei Wu

**Affiliations:** 1 Department of Psychiatry, National Clinical Research Center for Mental Disorders, The Second Xiangya Hospital, Central South University, Changsha, China; 2 Department of Psychiatry, University of Oxford, Oxford, UK

**Keywords:** Violence risk assessment, Prediction, Severe mental illness, Recidivism, Forensic psychiatry, FoVOx

## Abstract

**Background:**

Violence risk assessment is a routine part of clinical services in mental health, and in particular secure psychiatric hospitals. The use of prediction models and risk tools can assist clinical decision-making on risk management, including decisions about further assessments, referral, hospitalization and treatment. In recent years, scalable evidence-based tools, such as Forensic Psychiatry and Violent Oxford (FoVOx), have been developed and validated for patients with mental illness. However, their acceptability and utility in clinical settings is not known. Therefore, we conducted a clinical impact study in multiple institutions that provided specialist mental health service.

**Methods:**

We followed a two-step mixed-methods design. In phase one, we examined baseline risk factors on 330 psychiatric patients from seven forensic psychiatric institutes in China. In phase two, we conducted semi-structured interviews with 11 clinicians regarding violence risk assessment from ten mental health centres. We compared the FoVOx score on each admission (*n* = 110) to unstructured clinical risk assessment and used a thematic analysis to assess clinician views on the accuracy and utility of this tool.

**Results:**

The median estimated probability of violent reoffending (FoVOx score) within 1 year was 7% (range 1–40%). There was fair agreement (72/99, 73% agreement) on the risk categories between FoVOx and clinicians’ assessment on risk categories, and moderate agreement (10/12, 83% agreement) when examining low and high risk categories. In a majority of cases (56/101, 55%), clinicians thought the FoVOx score was an accurate representation of the violent risk of an individual patient. Clinicians suggested some additional clinical, social and criminal risk factors should be considered during any comprehensive assessment. In addition, FoVOx was considered to be helpful in assisting clinical decision-making and individual risk assessment. Ten out of 11 clinicians reported that FoVOx was easy to use, eight out of 11 was practical, and all clinicians would consider using it in the future.

**Conclusions:**

Clinicians found that violence risk assessment could be improved by using a simple, scalable tool, and that FoVOx was feasible and practical to use.

**Supplementary Information:**

The online version contains supplementary material available at 10.1186/s12888-021-03115-3.

## Background

Many population-based and cohort studies have reported a small but clinically important increased risk of violence in many mental disorders [1, 2], which is modifiable, and can lead to disruptions in care, increased stigmatization, and substantial healthcare costs associated with detention in secure hospitals [3, 4]. Violence risk assessment is considered one part of a multi-faceted approach to reduction of violence risks and can assist clinical decision-making about psychiatric admission, management, and discharge, and improving linkage to violence-reducing interventions such as optimization of medication, more frequent follow-up and additional psychosocial treatments [5]. Currently, however, many mental health professionals, including those in China, tend to rely predominantly on unstructured clinical judgment [6], partly due to lack of validated tools. A recent meta-analysis has suggested that existing violence risk assessment tools such as Historical, Clinical, Risk Management-20 (HCR-20) and Psychopathy Checklist Revised (PCL-R) have poor to moderate predictive validity in Chinese settings [7], which corresponds with poor validity in field studies in people leaving hospital and prison in other countries [8, 9]. Another limitation of current instruments has been that they are often resource-intensive and time-assuming. For example, an initial assessment using HCR-20 usually takes 15 person-hours [10], professional time that could be better directed towards treatments. Furthermore, current violence assessment tools have been developed on heterogeneous and non-psychiatric populations and were not developed using high quality methods [11]. A particular high-risk group are patients who have criminal histories, and on discharge from forensic (or secure) hospitals, reoffending risk is estimated at 4484 per 100,000 person-years according to a recent review [12]. Individual studies have found 6% reoffend within 6 months of discharge in Germany [13], and 49% repeat offend within around 3 years in England [14].

To address shortcomings with previous instruments, a new prediction rule (Forensic Psychiatry and Violence tool Oxford, FoVOx [15]) has recently been developed to estimate violent risk of patients following discharge from secure psychiatric hospitals. The FoVOx tool was developed using a longitudinal cohort study of 2248 patients in Sweden. This tool is quick and simple to use, and consists 12 routinely collected risk factors, including demographic (i.e. sex, age, and employment at admission), criminal (i.e. previous violent crime, previous serious violent crime) and clinical factors (i.e. primary discharge diagnosis, drug use disorder at point of hospitalization or discharge, any lifetime drug use disorder, alcohol use disorder at point of hospitalization or discharge, personality disorder at discharge). The development FoVOx study was conducted using high quality methods including a pre-specified protocol, transparent reporting of results, and translation into a freely-available online calculator (https://oxrisk.com/fovox/). The tool was internally validated and has a good predictive accuracy (area under the curve = 0.77 at 12 and 24 months). It remains to be externally validated, which will likely need a multi-country study due to the relatively small numbers of patients in forensic hospitals in any particular country. A recent study has showed that the tool can be adapted for use in prison hospitals in Germany with good performance in predicting inpatient violence [16]. Furthermore, there are very few studies of risk assessment tools in low and middle income countries and the feasibility of such approaches needs to be examined in such settings.

To further examine the utility of the FoVOx tool, we conducted a clinical impact study to assess: (i) the distribution of violence risk scores in a representative Chinese cohort of forensic psychiatric patients and (ii) the potential impact of the tool on mental health professionals in assisting their decision-making using qualitative methods. This study, thus, primarily aims to test acceptability and potential utility of using one standardised risk assessment tool in Chinese psychiatric settings.

## Methods

### Study design and participants

We used a mixed-methods approach to examine the feasibility of FoVOx (Supplemental materials, Figure 1). This study includes two phases. In phase 1, we retrospectively examined patients who had committed violent crimes and admitted for forensic psychiatric assessments between 1 January 2011 and 31 December 2011 in seven forensic psychiatric institutes in Hunan Province, China. We manually reviewed all medical files of patients under forensic psychiatric assessments in Hunan to extract data on variables included in the FoVOx risk assessment tool. We included patients who were (i) ≥18 years old; (ii) diagnosed with mental illness following the criteria of the International Classification of Diseases 10th Revision; (iii) Hunan residents; and (iv) suspected of a violent offence. We excluded patients who were diagnosed after crime dates.

In phase 2, we used a convenience sampling method to recruit clinicians from ten mental health centres and conducted in-depth interviews using a semi-structured interview questionnaire between 1 May 2019 and 31 December 2019. We included clinicians who had experience with violent risk assessment. Based on a purposive sampling procedure and principle of data saturation, we interviewed 11 clinicians for their opinion on the impact of the tool. Unlike external validations, currently there is no consensus on the minimum number of participants required for qualitative feasibility studies [17]. However, it has been suggested that a sample size of ten is sufficient to identify potential issues of a tool [18].

### Risk factors

Two groups of risk factors were considered based on whether they were presented in the original model in FoVOx (see: https://oxrisk.com/fovox/). Group 1 risk factors includes four broad classes of variables: social-demographic factors (sex, age, employment before admission), criminological factors (any previous violent crime, any previous serious violent crime), clinical factors (inpatient episodes, length of stay), and psychiatric diagnosis (primary discharge diagnosis, drug use disorder at hospitalization or discharge, lifetime drug use disorder, alcohol use disorder at hospitalization or discharge, personality disorder diagnosis at discharge).

In this study, we defined ‘full-time students’ as ‘employed’ and ‘farmers’ as ‘unemployed’ to reflect the local context (as farmers are typically in insecure and irregular employment). We did not include one variable (length of stay in forensic hospital) as it is not applicable in this study setting (i.e. the patients were to be transferred to forensic hospitals at the time of assessment).

Group 2 were proxy variables which were approximate to the original variables in FoVOx and were associated with outcomes. For example, any previous diagnosis of drug use disorder was indicated with a comparable variable, history of drug use; any previous diagnosis of alcohol use disorder was indicated with history of alcohol use. History of alcohol use and drug use was adopted as proxy factors for lifetime alcohol use disorder and lifetime drug use disorder, respectively.

### Semi-structured interview survey

We developed a standardized tool to collect data from clinicians. The interview questionnaire was reported in a previous study [19] and was translated. We explored the following domains to examine the feasibility of FoVOx in clinical settings, including:

- Accuracy (i.e. Do you think that the FoVOx score was an accurate representation of the risk that this patient posed at the time of assessment?);
- Impact (i.e. Do you think that knowing the patient’s FoVOx score at the point of assessment would have been of any clinical benefit, e.g. would it have altered your management of this patient?);
- Practicability (i.e. Having seen the tool, do you think that the FoVOx web-based calculator would be practical to use as part of a discharge assessment/plan?);
- Simplicity (i.e. Do you think that it would be possible to complete the FoVOx tool without having to refer to the clinical notes in the majority of cases?);
- Future use (i.e. Will you use FoVOx in the future?).

### Procedures

In phase 1, as part of the forensic psychiatric assessments, the current episode and clinical diagnosis were assessed by a minimum of two forensic psychiatrists. Information (demographic data, medical records and criminal files) were collected from medical records. These were also used to collect the history of medical factors (inpatient episodes, length of stay). All data were retrieved by an independent researcher (CL) using a standard form. Then, the data were evaluated by a second researcher (SZ) for eligibility.

In phase 2, before the interview, clinicians were asked to complete FoVOx for up to 10 patients that they had assessed. During the interview, clinicians were asked to estimate the violence risk level (low/medium/high) of their patient before they saw the FoVOx score. Then, they were given the FoVOx risk score of the same patient (which is a probability score ranging from 0 to 20%). Afterwards, clinicians were asked whether they considered the FoVOx score was accurate for this particular patient and whether there was any clinical benefit of knowing the FoVOx risk category and probability score at the point of assessment. Finally, clinicians were asked whether the tool was practicable and simple to use and whether they would use FoVOx in the future.

### Outcomes

The primary outcomes were predictor information on the patient cohort, FoVOx scores of the patients, and clinicians’ responses to the qualitative questionnaire.

### Analytic strategies

In the quantitative analyses, we calculated the frequency of risk factors for categorical data and mean (standard deviation) for continuous data. To explore the feasibility of collecting the required data, we recorded the proportion of missing data and coded them as ‘unknown’. We calculated agreement in risk assessment categories (i.e. low/medium/high) between the judgement from the clinicians and the FoVOx score. We combined medium and high-risk categories and compared it to the low risk category, and calculated *Cohen’s* kappa [20]. As an alternative approach, we also calculated kappa to compare low with high-risk categories.

In qualitative analyses, we recorded semi-structured interviews with clinicians. We made notes during the interview and used their original words as many as possible, and also reported back the interpretation of their views to give them an opportunity to confirm or amend this. SZ analysed the records, coded items and then grouped items into separate themes. A second researcher (RC) repeated the coding independently and the two researchers reached a consensus on the data interpretation.

## Results

### Sample

In the first phase, 330 patients were included (17.6% females), with a median age of 36 years (ranging from 25 to 47). In phase 2, we identified 110 admissions for psychiatric assessments in forensic psychiatric hospitals. Nine patients were excluded, as five of them did not have mental illness at the time of the index crime, three had unclear information on risk factors, and one had not committed any crime. In total, data from 101 patients were analysed. The number of assessed patients per clinician ranged from 6 to 10. Among 11 clinicians interviewed, 9 reported that they did not use any risk assessment tool but adopted a standard review of clinical and criminal reports and clinical interviews. Only one clinician reported using a risk assessment tool that was developed locally by the hospital and one clinician reported using HCR-20 items.

### Baseline characteristics and risk factors

The distribution of the risk factors in this cohort and comparisons with the development one are shown in Table 1. Most patients were male (82.4%), unemployed (87.3%), had a history of violent crime (62.4%), and had a diagnosis of schizophrenia-spectrum disorders (67.9%).

**Table 1:** Distribution of risk factors for violence in severe mental illness in Hunan and in comparison with the original Swedish cohort

Variables	Hunan (*N* = 330)		Sweden (*n* = 2248)	
Age (IQR)	36	25–47	36	29–45
	n	%	n	%
Gender				
Male	272	82.4	1938	86.0
Female	58	17.6	310	14.0
Employment				
Employed	27	8.2	171	7.6
Unemployed	288	87.3	2077	92.4
Any previous alcohol use disorder				
Yes	–	–	780	34.7
Any previous drug use disorder				
Yes	–	–	1050	49.0
Previous violent crime				
Yes	206	62.4	1836	81.7
Previous inpatient episodes				
≥5	8	2.4	1110	52.6
3–4	25	7.6	–	–
1–2	90	27.3	–	–
0	10	3.0	–	–
Primary diagnosis				
Schizophrenia-spectrum disorders	224	67.9	944	45.7
Bipolar disorder	29	8.8	130	6.3
Unipolar depression	0	0	97	4.7
Anxiety disorders	2	0.6	139	6.7
Other	75	22.7	754	36.5
Drug use disorder at hospitalization or discharge				
Yes	16	4.8	540	26.2
Alcohol use disorder at hospitalization or discharge				
Yes	9	2.7	217	10.6
Personality disorder at discharge				
Yes	4	1.2	536	27.3

### FoVOx scores

We calculated FoVOx scores based on data from the clinical recodes for each patient included at the phase 2 and also asked clinicians for their risk rating (Table 2). Based on FoVOx, the median probability of violent reoffending within 2 years was 7% (range from 1 to 40%). As for risk categories, 17 (16.8%) of individuals were categorized as low risk, 76 (75.2%) medium risk and 6 (5.9%) high risk based on pre-specified thresholds.

**Table 2:** Risk categories assigned by clinicians compared with categories based on FoVOx scores

	FoVOx	
	Low	Medium/High
Clinicians		
Low	8	18
Medium/High	9	64

FoVOx risk categories are based on pre-specified risk levels. Low: < 5%; medium: 5–20%; high:> 20%

### Concordance between FoVOx and clinical judgement

We calculated concordance between the risk levels (i.e. low/medium/high) obtained from the FoVOx in relation to clinical judgement in individual patients. For 2 individuals, clinicians reported unable to provide the risk category, and the comparison was not made. We found agreement between risk categories based on FoVOx and those from clinicians in 72% of cases (72 out of 99, 73% agreement, kappa = 0.21 indicating fair agreement). For cases with disagreement between risk categories, clinicians tended to score patients at a lower risk (18 out of 27 the cases) than the FoVOx category (9 out of 17). When using two risk bins (low and high), 83% (10 out of 12) cases were agreed between FoVOx and clinicians (83% agreement, kappa = 0.57, indicating moderate agreement).

For the majority of cases (56/101, 55.4%), clinicians thought that the FoVOx score was an accurate representation of the violence risk. In 14.9% of cases (15/101), clinicians were unable to comment about FoVOx accuracy. In 30/101 (29.7%) of cases, clinicians thought that FoVOx was not wholly accurate and were asked to give reasons. They identified the main reason as being that certain individual risk factors were not included in FoVOx and summarised these into three groups (Table 3). First, clinicians stated that certain clinical risk factors were not identified, such as a family history of mental illness, psychotic symptoms, adherence with medication, and response to treatment. These factors were thought to contribute to risk in both directions: if the patient has a poor adherence or partial response to medication, the FoVOx score might be an underestimate and vice versa. “*The characteristic of the disease is not identified in the tool. In this case, the patient has a poor response to treatment*”. “*The FoVOx score is high in this case. However, the patient has good medication compliance*”. A second group of possible missing factors were social ones, including family supervision, social support and social status. “*The score rated by the tool was low. But this patient may still be quite aggressive, as he has a strong family history of mental illness and poor social support*.” Finally, clinicians reported that more criminal risk factors should be considered, including the individual’s attitude towards the violent crime committed, recent violent behaviour in hospital, and instrumental reasons for the violence. “*The patient had this violence incident for a certain realistic reason. The victim owed money and did not pay it back*”.

**Table 3:** Viewpoints on what other individual and social factors are relevant for violence risk assessment (case by case)

Theme	Sub-theme	FoVOx score higher than clinical judgement as the tool does not include the following protective factors	FoVOx score lower than clinical judgement as the tool does not include the following risk factors
**Social risk factors**	supervision	good family support	poor family support (no close family, anger towards family)
	social support	good social support	poor social support
	social status	had an official job	homeless; low social status
**Clinical risk factors**	family history	–	history of mental illness in family members
	disease related	–	active and positive symptoms; poor insight; chronic disease; early onset age
	treatment related	good response to medication; good compliance	poor response to medication; poor compliance; not receiving systematic treatment
	diagnosis related	only had history of psychiatric diagnosis, no recurrence afterwards	high impulsivity; low self-control
**Criminal risk factors**	response to the violent crime committed	–	not feeling guilty or regretful
	previous violent history	–	history of repeated violence; frequent recent violent behavior in hospital
	individual factors	triggers/underlying reasons for crime may have been resolved	short length of imprisonment triggers/underlying reasons for crime had not been resolved

### Viewpoints on utility at the point of admission

We asked clinicians if it is beneficial to know FoVOx scores at the point of first assessment. Clinicians reported that it would be helpful in 52 (50%) out of 101 cases. A summary of the qualitative feedback is presented in Table 4. We identified two themes. First, clinicians viewed that the FoVOx tool can assist clinical decision-making. Clinicians frequently mentioned that it can be used as part of patient management, especially for cases with a high risk. “*In a high-risk case like this, I would suggest compulsory medical treatment in the final report, and would refer to the tool, including the probability value from the tool*”. It was reported that using the FoVOx score can facilitate liaison with third parties without a professional background. “*The scores are intuitive and easy for people without a professional background to understand. They could be used as a basis for communicating with patients*”. It may also improve information sharing among medical staff. It was reported that for individuals with a low or medium risk, no difference would be made in management decisions as majority of them would be sent to prisons rather than forensic hospitals. For these cases, “*the aim at the time of assessment is mainly to estimate the current violent risk rather than future risk.”*

**Table 4:** Viewpoints on utility at the point of admission (case by case)

Themes	Sub-theme	Helpful	Not helpful
**Assist decision making**	As part of disposal suggestions	For high-risk individuals, clinicians will refer to the tool and make suggestions on compulsory treatment to guide disposal for the law enforcement agencies.	No difference in disposal decision for low- or moderate-risk individuals: patients will go to prisons.
	ln liaison with third parties	Help to explain to people without a professional background using a more objective tool, e.g. patients, family members, the police	Other agencies care about categorical risk levels (e.g. high/low) rather than probability scores.
	ln liaison with other clinicians	Consistent risk levels; able to communicate among clinicians and nurses.	
**Impact on risk assessment**	As part of clinical plan and management	Add supportive information, and possible to choose a more conservative risk assessment plan	If there is a difference between: FoVOx scores and clinical judgement, clinicians might revert to clinical judgement.
	Reassurance	Confirm and support clinical assessment if the risk levels between clinicians and FoVOx are consistent	
	Existing perceptions of risk assessment	The FoVOx score provides an objective view	The tool has not been externally validated yet; Not sure how to use probability value. Not clear from online calculator how risk factors are weighted.
	Reminders for added risk factors	Clinicians would consider other risk factors if FoVOx has a higher value and would consider protective factors if FoVOx has a lower value than clinician judgement	Not many clinical and dynamic factors

The second theme was how the FoVOx tool could impact on individual risk assessment. Some clinicians reported that FoVOx could be used as part of clinical management and may support clinical judgement. Others said that the FoVOx tool would not contribute if it differed from clinical judgement. Some clinicians noted that it can serve as a reminder to consider additional risk factors: when FoVOx has a higher value than the clinicians’ judgement, they would consider if there are additional risk factors; and when FoVOx has a lower value than clinicians’ judgement, they would consider if there are other protective factors. A very few found that the tool may not be as helpful as insufficient risk factors were included in the tool.

### Overall views of practicality and future use

After showing the clinicians the FoVOx tool, 10 out of 11 (91%) clinicians reported that it would be possible to complete the FoVOx score calculation without having to refer to the clinical notes in majority of cases. One clinician noted that it would be safer to refer to additional information. Three reported that the FoVOx tool had practical difficulties. We identified two themes noted by these clinicians: some risk factors were not included in the tool and some risk factors needed further adjustment (see Table 5 for a summary).

**Table 5:** Viewpoints on practicality and future use of FoVOx risk assessment tool

Themes	Sub-theme	Reasons
**Will use in the future**	FoVOx specific	simple items, easy to measure; quick, convenient and free calculator; important risk factors included; reliable, consistent with clinical judgment; well developed and will use it if further validated; rather objective
	Need for tools	need for a standardized tool; there is no tool currently
	Information sharing	provides informative data to support; help to explain to judges
	Impact on risk management	reassurance with clinical judgements; easy to refer using low/middle/high categories
**Limited use**	Risk factors not identified	only a few items were included; the risk factors are not associated with high variance
		numbers of violence incidence, incentives for violence, family support, education level, current symptoms, medication adherence, duration of disease
	Risk factors need adjustment	Few diagnoses of personality disorders are made in China. It is difficult for judges to understand. There is no special treatment for PD, and may increase the stigma towards the individual with PD.
		Data on diagnosis of drug abuse is not commonly collected in China.

All 11 clinicians reported that they would use FoVOx in the future. We identified two main themes (Table 5): there is a need for tools in China and the advantages of FoVOx. “*At present, there is no such tool in China, and the variables included in the (FoVOx) tool are really important. If the tool is further verified, it can be used as a supplement (in risk assessment/management plan). But to be more cautious, other factors should be considered as well*”.

## Discussion

We completed the FoVOx risk assessment tool on 330 patients who had committed a crime and had undertaken a full psychiatric assessment in one province in China. We then, based on a sample of another 110 patients from different provinces, assessed the perceived accuracy, usefulness and feasibility of the FoVOx tool by conducting in-depth interviews with clinicians.

We found that most items contained within the FoVOx tool could be identified quickly and by clinician recall. Some items needed modification, and there are differences between forensic psychiatric populations in Sweden and China. Although age, gender and employment at the point of admission were similarly distributed, patients in China were less likely to have committed a previous violent crime (62.4% vs 81.7%), and much less likely to have had multiple admissions to psychiatric hospital (2.4% having had five or more admissions vs 52.6%). Patients in China were more likely to have a primary diagnosis of schizophrenia, and personality disorder was less common. Substance use disorders, both for alcohol and drugs, were less prevalent in the Chinese sample. These differences in prevalence do not lead to more inaccurate risk scores assuming the effect of each risk factor remains similar.

Despite these differences in the prevalence of risk factors, the FoVOx tool was considered acceptable by clinicians, with interviews suggesting strengths including its ability to assist with clinical decision-making and increase transparency among risk assessments conducted by different clinicians. In addition, the online risk score output includes easily understandable information which could facilitate communication with other agencies. Furthermore, the tool may help to identify risk factors not identified in unstructured clinical assessment.

Qualitative analysis found that, in some cases, clinicians felt that FoVOx scoring was limited as it did not account for certain individual factors. Some of these factors may however be measured indirectly by FoVOx. For example, close supervision was considered by clinicians to be missing for some cases as a factor reducing risk [21]. We did not include the item on length of stay for patients as they were being scored at the time of transfer to hospital rather than discharge, but this may be overlap with length of stay variable. Normally, patients with a length of stay of over 1 year are likely to have increased levels of monitoring after discharge. Also, some of these perceived omissions do not actually have an impact on the risk of recidivism. However, some of them do, such as high levels of acute psychopathology and poor adherence to treatment [22]. These factors are harder to measure in a straightforward binary way, and are liable to unreliable measurement. Such factors will also change dynamically over time and depend on the other matters, such as level of insight and the level of treatment compulsion based on their legal status. One solution may be to combine FoVOx scoring with other dynamic risk assessment tools [23] that provide serial monitoring of risk.

Another main finding is the absence of currently used violence risk assessment tools in China. Of 11 participants, only two currently used a formal risk assessment tool. There are significant variations in risk assessment practice worldwide, with professionals in Asia using risk instruments less frequently than in Europe and North America [24]. Thus there is a need for a freely available, transparent, scalable, and well-designed risk assessment tools for use in China and other countries at similar points in the development of their mental health services, such as other middle-income countries in East Asia.

Another implication is that the agreement level between the judgement from the clinicians and the calculation from FoVOx was different when three risk categories (i.e. low/medium/high) and two risk categories (low and high) were used. A different approach would be to allow clinicians and researchers to maximize either sensitivity or specificity depending on the different expected prevalences of the outcome, although this would require careful collection of historical data.

### Limitations

Although seeking to understand the acceptability and utility of predictive tools is important, this is one part of a wider picture that needs to be considered as part of any implementation strategy. Another is external validation where a cohort is followed up over 1–2 years and information on outcomes collected. This would complement the present feasibility study, which focuses on useability and clinical utility. This should be pursued, although there are significant challenges in doing this in a forensic psychiatric population, given the large number of cases required. A large multicentre trial would be required in order to do this, and the feasibility of this will need to be determined.

Some items within the FoVOx tool may need to be adapted to suit the local characteristics. For example, personality disorder was felt to be present in a very small number of cases in this sample. Such diagnoses are likely to be more prevalent than we reported as we relied on medical records, albeit probably at a lower frequency than in Western European and North American samples [25]. Qualitative feedback suggested that there may be a reluctance to make personality disorder diagnoses due to fear of stigmatisation. Hence, this item could be altered as to whether the patient has significant personality traits, rather than basing it on a formal diagnosis. Similar changes could be made to items assessing substance misuse, as formal diagnoses relating to substance use are less likely, and in this study, we used proxy measures for these variables.

## Conclusions

Although developed in Sweden, we found that FoVOx was feasible and practicable to be used in a different country, China, with evidence that it could potentially impact patient management. Given that the tool relies on replicated risk factors, supported by a clear evidence base, most of which are categorised in a simple dichotomous way, FoVOx can be used in a minimally resource-intensive manner. Furthermore, the tool’s scalability, transparency and easily understandable outputs underscore its potential to inform clinical care in high risk patient groups.

## Supplementary Information

**Additional file 1: Figure S1.** Flow-diagram of the study design.

## Data Availability

The datasets used and/or analysed during the current study are available from the corresponding author on reasonable request.
