# Correction to: Assessment of violence risk in 440 psychiatric patients in China: examining the feasibility and acceptability of a novel and scalable approach (FoVOx)

**DOI:** 10.1186/s12888-021-03183-5

**Published:** 2021-04-09

**Authors:** Shaoling Zhong, Rongqin Yu, Robert Cornish, Xiaoping Wang, Seena Fazel, FoVOx group

**Affiliations:** 1 Department of Psychiatry, National Clinical Research Center for Mental Disorders, The Second Xiangya Hospital, Central South University, Changsha, China; 2 Department of Psychiatry, University of Oxford, Oxford, UK

**Correction to: BMC Psychiatry 21, 120 (2021)**

**https://doi.org/10.1186/s12888-021-03115-3**

Following the publication of the original article [1], the authors identified an error in the author name of Robert Cornish. The given name and family name were erroneously transposed.

The incorrect author name is: Cornish Robert

The correct author name is: Robert Cornish

The author group has been updated above and the original article [1] has been corrected.
