# Supplementary material for: Assessment of violence risk in 440 psychiatric patients in China: examining the feasibility and acceptability of a novel and scalable approach (FoVOx)
Source: BMC Psychiatry. 2021 Mar 2;21:120. doi: 10.1186/s12888-021-03115-3 (PMC7923307; doi:10.1186/s12888-021-03115-3)
Supplement: Supplementary file 1 — Additional file 1: Figure S1. Flow-diagram of the study design. [file 12888_2021_3115_MOESM1_ESM.docx]

Supplemental materials:

**Figure 1. Flow-diagram of the study design**
